# Concussion knowledge among family physicians in Croatia

**DOI:** 10.2217/cnc-2018-0002

**Published:** 2018-11-19

**Authors:** Marko Herceg, Linda Lusic Kalcina, Ivo Lusic

**Affiliations:** 1 Phelps Hospital, Northwell Health, Sleepy Hollow, NY 10591 USA, School of Public Health, New York Medical College, Valhalla, NY 10595 USA; 2 Department of Neuroscience, University Hospital of Split, University of Split School of Medicine, Soltanska 2, 2100, Spilt, Croatia; 3 Department of Neurology, University of Split School of Medicine, Soltanska 2, 2100, Split, Croatia

## Abstract

**Aim:**

The objective of this study was to administer and analyze results of a survey targeting knowledge about concussion symptoms, diagnosis, treatment and expected recovery among family medicine specialists in the Split–Dalmatia County of Croatia.

**Methods:**

An electronic survey questionnaire was developed utilizing concepts from previously published studies on concussion knowledge, attitudes and beliefs completed by physicians. The survey was intended to briefly and broadly assess concussion knowledge of Croatian healthcare providers. The first section of the survey included five questions clarifying professional practice, years of experience and experience with concussions; the second section included 15 questions about typical concussion symptoms; the third section included 12 questions focused upon three primary components of concussion knowledge: concussion diagnosis, treatment and recovery.

**Results:**

Out of 242 surveys mailed, 81 questionnaires (33%) were completed while 161 respondents (67%) did not answer. Out of the 81 completed surveys, 76 (94%) were returned by family physicians specialist and five (6%) by resident physicians in training. 39 (48%) had treated less than ten patients with concussion during last year: 40 (49%) treated 11–20 patients with concussion; and two (3%) treated greater than 20 patients with concussion during last year. While most responses did accurately reflect knowledge of common symptoms (90–100% correct), there was significant lack of knowledge in three areas: only 19% of participants stated that diagnosis of concussion does not require loss of consciousness; three quarters of respondents believed that a diagnosis of concussion requires direct contact to the head and 83% of the respondents believed that persistent subjective complaints are always the result of a more severe initial injury.

**Discussion:**

This is the first investigation conducted in Croatia to examine knowledge of concussion diagnosis, as well as the management practices held by medical professionals. Overall, the findings suggest that the knowledge and management practices among family doctors in the region are not consistent with current worldwide views and recommendations. There was not an accurate knowledge of concussion diagnosis, treatment, recovery and prognosis among family physicians. Continued education of medical staff to better identify concussion and increased reliance on objective methods for managing concussion will improve patient management and outcome.

In November 2001, the first International Symposium on Concussion in Sport was held in Vienna, Austria. The aim of the symposium was to bring experts together, providing recommendations for the improvement of safety of athletes who suffer concussive injuries in contact or collision sports. The conference was attended by a wide range of international experts. The goal was to address specific issues of epidemiology, basic and clinical science, grading systems, cognitive assessment, research methodology, protective equipment, management, prevention and long-term health outcome. At the conclusion of the conference, a small group of experts were given a mandate to draft a document describing the position statement reached by those in attendance at that meeting [1]. Five subsequent meetings have been held, further examining and clarifying concussion from diagnosis, to treatment, to epidemiology. The most recent consensus meeting was held in Berlin in October 2016 [2].

Since 2001, there has also been an abundance of information on sports concussions and additional position statements from various sport and medical organizations (AAN) as well as governments (CDC and Canada). Yet surveys of healthcare providers still continue to find that barriers to the successful management of sports concussion remain [3].

In USA, the general trend is that pediatric primary care, sports medicine and emergency medicine providers regularly care for concussion patients. Some studies have found that these groups may not have adequate training or infrastructure to systematically diagnose and manage these patients [4]. In addition, many providers are unfamiliar with, or do not use, published concussion guidelines and report varying practices in treatment of concussion patients. One study demonstrated that there is a need for further education for pediatric providers who see patients with concussion [5].

In Canada, one study found substantial gaps in knowledge surrounding concussion diagnosis and management among family medicine residents [6]. Other studies have demonstrated a clear gap in concussion knowledge among neurology and neurosurgery residents [7], as well as gaps in the knowledge of emergency medicine physicians and family medicine physicians [8]. In a study conducted by Ryu *et al*. [9], expert review of medical charts found deficiencies in concussion diagnosis and management by primary care physicians.

If knowledge of concussion in advanced and developed countries is lacking, then it is not surprising that a study in Sri Lanka found that few emergency and surgical physicians are aware of concussion management guidelines and continue to follow outdated advice [10].

When examining these studies, there appears to be inadequate health literacy and knowledge translation regarding concussion among physicians.

Since obtaining independence in 1991, Croatia as a nation has had many sports achievements. Notably, the men's Silver Medal in basketball at the 1992 Summer Olympics in Barcelona, reaching the FIFA World Cup semi-finals in 1998 and finals in 2018, just to name a few. In addition, many of its top athletes have gone on to play at the highest level in European Club Soccer, the NBA and medal in both the Summer and Winter Olympics. With the increase in knowledge and awareness of sports-related concussions, combined with a rich history of sports achievements, the First Croatian Conference on Sports Related Brain Injury was held at the University of Split Medical School, 15–17 June 2017. The conference was the first of its kind in the region, and ten international experts attended and presented the latest science and current guidelines. As preparation for the conference, this study was conducted. This was the first to investigate knowledge and attitudes about concussion diagnosis and management among family medicine physicians.

## Methods

An electronic survey questionnaire was developed utilizing concepts from previously published studies on concussion knowledge, attitudes and beliefs. The survey was intended to briefly and broadly assess concussion knowledge of Croatian healthcare providers.

The first section of the survey examined scope of practice. This included years of experience in current role, current place of work (urban vs. rural); years of education in sports medicine: formal training in concussion care; and experience of work with concussion cases.

The second section included 15 questions about typical concussion symptoms; nausea, appetite, dizziness, hyperactivity, sleep, excitability, headache, mental daze, impatience, difficulty concentrating, nervousness, difficulty hearing, feeling slowed down, talking more and confusion.

The third section included 12 questions that focused on three primary components of concussion knowledge: concussion diagnosis, treatment and recovery.

The survey was disseminated via a provider e-mail list directory to providers in the Split–Dalmatia County. The email lists were maintained and generated at the University of Split Medical School and respondents were able to complete it anonymously via a Survey Monkey platform.

## Results

242 surveys were e-mailed, 81 (33%) were completed while 161 respondents (67%) did not respond.

Out of the 81 completed surveys, 76 (94%) were returned by family physicians specialists, and five (6%) were by resident physicians in training.

With regard to professional experience in the role of family medicine physician, 21 (26%) had less than 10 years; 47 (58%) had 11–25 years and 13 (16%) had over 25 years of experience in the medical field (Table 1).

**Table 1.** **Years of practice in family medicine.**

**Years of practice**	**Number of physicians**
Less than 10 years	21 (26%)

11–25 years	47 (58%)

Over 25 years	13 (16%)

Median: 21 years (range 2 to 37).

47 (58%) stated they practice in urban areas while 34 (42%) were in rural. 12 (15%) stated they had education and training in sports medicine while 69 (85%) did not have any formal training. No provider reported any formal training in concussion care (Table 2).

**Table 2.** **Work experience, training and education.**

**Place of work**	**Response**
Urban	47 (58%)

Rural	34 (42%)

Education in sports medicine?	

Yes	12 (15%)

No	69 (85%)

Formal training in concussion?	

Yes	0

No	81 (100%)

During the course of the past year, 39 (48%) stated they treated less than ten patients with concussion; 40 (49%) treated 11–20 and only two (3%) treated greater than 20 patients who sustained concussions.

As evidenced in Table 3, almost all providers appeared to have a good knowledge of physical and/or cognitive signs and symptoms, but less about behavioral ones.

**Table 3.** **General knowledge of concussion.**

	**Symptom/sign**	**True**	**False**	**Percentage of correct answers**
**1.**	**Confusion**	**81^†^**	0	100%

**2.**	**Headache**	**80^†^**	l	99%

**3.**	**Difficulty concentrating**	**77^†^**	4	95%

**4.**	**Nausea**	**77^†^**	4	95%

**5.**	**Dizziness**	**75^†^**	6	93%

**6.**	**Slowed down feeling**	**75 ^†^**	6	93%

**7.**	**Mentally dazed**	**73^†^**	8	90%

**8.**	**Sleep more**	**73^†^**	8	90%

9.	Eat more	8	**73^†^**	90%

10.	Talk more	9	**72^†^**	89%

11.	Impatient	14	**67^†^**	83%

12.	Hyperactive/high energy	21	**60^†^**	75%

13.	Excited	33	**58^†^**	72%

14.	Nervousness	45	**36^†^**	44%

15.	Difficulty hearing	54	**27^†^**	33%

Bold font represents those responses which were appropriate and acceptable.

^†^Above acceptable thresholds.

General knowledge of concussion (Table 4) revealed a wider discrepancy in knowledge, particularly regarding mechanism of injury, severity of injury, loss of consciousness and risk for increased injury and neuropathological changes.

**Table 4.** **Survey findings.**

	**Statement**	**True**	**False**	**Percentage of correct answers**
1.	A concussion is usually associated with normal CT/MR scan	**77^†^**	4	95%

2.	A youth (18 years) who has had loss of consciousness can return to full activities in the same day once the concussion symptoms are resolved	5	**76^†^**	94%

3.	After a recent concussion, a person has a greater risk for another concussion	**71^†^**	10	88%

4.	A concussion sustained in youth (≤18 years) should be managed more conservatively compared with a concussion sustained in adulthood (18≥ years)	**67^†^**	14	83%

5.	Resolution of concussive symptoms at rest is sufficient to allow a youth (≤18 years) with a concussion to return to full activities without further testing	17	**64^†^**	79%

6.	During the first few days after a concussion, best practices of care include outpatient therapies (e.g., physical therapy, occupational therapy, etc.)	20	**61^†^**	75%

7.	Problems related to a concussion always begin immediately after the concussion	39	**42^†^**	52%

8.	A single concussion increases the risk for chronic traumatic encephalopathy	51	**30^†^**	37%

9.	Is chronic traumatic encephalopathy an established medical condition that can be diagnosed?	58	**23^†^**	28%

10.	A concussion requires contact to the head	61	**20^†^**	25%

11.	A concussion requires loss of consciousness	66	**15^†^**	19%

12.	Patients who have concussion-related complaints months later usually experienced a more severe injury	67	**14^†^**	17%

Bold font represents those responses that were appropriate and acceptable.

^†^Above acceptable thresholds.

The vast majority of respondents were correct that recovery does not end immediately after withdrawal of the symptoms. One quarter of respondents endorsed the benefit of multidisciplinary care in the initial days after a concussion, and 83% of participants recognized that younger athletes may take longer than adults to recover after. Almost half of respondents believed that the onset of symptoms is immediately after concussion.

## Discussion

This is the first investigation conducted in Croatia that examined knowledge of concussion diagnosis, as well as the management practices of physicians. Overall, the findings suggest that knowledge and management practice among family doctors in the region is not consistent with current international consensus, guidelines and recommendations. Although the response rate was low, considering the fact that this was the first ever survey, the rate was considered adequate.

Of particular interest, and concern, was that 25% (n = 20) of surveyed family medicine physicians still believe that direct trauma to the head was required to sustain a concussion and 19% (n = 15) stated that a loss of consciousness was required for diagnosis. This lack of knowledge and perception has potential ramifications as it might lead to underdiagnosis, mismanagement and poor outcome.

Thus, the present study reveals gaps in resident knowledge about concussion that reflect a need for improvement in medical education and training. What is most concerning, but not altogether surprising, is that all respondents reported not having had formal education or seminars about concussion and could not remember learning about concussion in their medical education. Moreover, none of the residents reported learning about concussion in their current residency training. This study reveals important opportunities for improvement in concussion teaching both in medical education and residency training programs.

## Limitations

The sample size in this study is small (n = 81; 33% response rate) but in fact considered adequate. In addition, this was a regional survey that was not sent to two other large urban centers, Zagreb and Rijeka, which have medical hospitals and rehabilitation centers. It is possible that those surveyed are not a representative sample of family medicine in Croatia. Thus, we were unable to directly compare the characteristics of our sample to the rest of the Croatian medical community.

## Conclusion

Overall, the findings suggest that despite formal training, the general knowledge of concussion symptoms was pretty high. The incorrect answers regarding chronic traumatic encephalopathy suggest important areas for research, education and management of concussions. This is especially important since Croatia's national sport is soccer, and there have been some studies demonstrating changes to the brain after heading the ball [10]. In addition, the lack of knowledge regarding loss of consciousness and head contact provides needs to be primary in education efforts in the future.

Concussion education targeted to medical professionals, but also to all professionals involved in sports activity, is key to increased concussion symptom reporting, and ultimately reduction of the potential for undiagnosed and untreated brain injuries [11]. The findings of this study, however limited, provide information and indicate that improvement in knowledge and awareness about concussion in Croatia is needed. We hope that this study, along with the recent First Croatian Conference on Sports Related Brain Injuries, can be the impetus to advance education, knowledge and awareness of concussion to those involved with athletes of all ages.
